# Supplementary material for: Measuring the Air Quality Using Low-Cost Air Sensors in a Parking Garage at University of Minnesota, USA
Source: Int J Environ Res Public Health. 2022 Nov 18;19(22):15223. doi: 10.3390/ijerph192215223 (PMC9690026; doi:10.3390/ijerph192215223)
Supplement: Supplementary file 1 [file ijerph-19-15223-s001.zip › ijerph-1981265-supplementary.pdf]

Supplementary Information

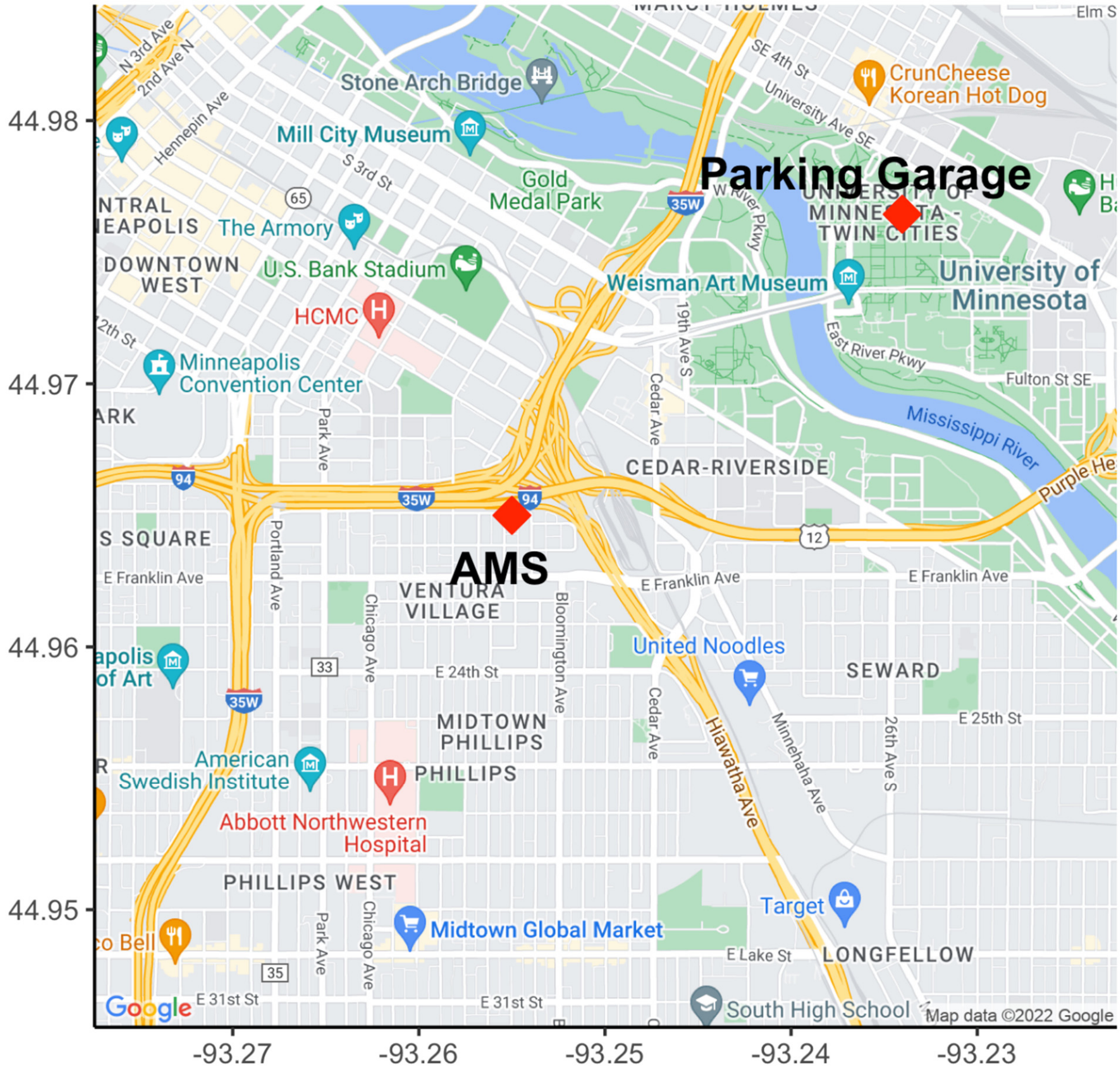

Figure S1. Location of the parking garage in East bank campus at University of Minnesota and MPCA air monitoring station (AMS).

Table S1. Daily average temperature and humidity from sensors and Partector

Day	Temperature °C	Temperature Partector °C	Humidity	Humidity Partector
24-Jul	34	31	42	43
25-Jul	34	32	44	44
26-Jul	34	31	56	54
27-Jul	34	31	50	49
28-Jul	34	31	56	54
29-Jul	34	31	48	48
30-Jul	33	30	34	36
31-Jul	33	30	38	39

Table S2. Overall p-values of the correlations among variables in study

	CO ppm	CO_2 ppm	NO ppb	NO_2 ppb	NOx ppb	O_3 ppb	LDSA $\mu m^2/cm^{-3}$	PM2.5 $\mu g\ m^{-3}$	Total Traffic In	Total Traffic Out
CO ppm	1									
CO_2 ppm	1.8E-75	1								
NO ppb	8.7E-165	3.7E-53	1							
NO_2 ppb	9.0E-20	5.6E-04	1E-13	1						
NOx	2.9E-165	1.4E-52	0E+00	3.2E-15	1					
O_3 ppb	2.9E-54	1.2E-35	1E-39	9.5E-30	1.2E-40	1				
LDSA	8.0E-71	7.7E-31	1E-55	5.0E-23	2.0E-56	3.2E-22	1			
PM2.5	1.2E-07	1.8E-10	4E-02	7.7E-01	4.9E-02	1.1E-02	9.8E-24	1		
Total Traffic In	3.0E-03	2.5E-10	5E-02	6.4E-02	4.6E-02	5.8E-05	7.6E-03	0.09	1	
Total Traffic Out	1.1E-62	6.2E-30	3E-39	1.2E-11	2.5E-39	3.1E-19	8.2E-31	0.00	0.004	1
